# Pathophysiological Roles of Transient Receptor Potential (Trp) Channels and Zinc Toxicity in Brain Disease

**DOI:** 10.3390/ijms24076665

**Published:** 2023-04-03

**Authors:** Dae Ki Hong, A Ra Kho, Song Hee Lee, Beom Seok Kang, Min Kyu Park, Bo Young Choi, Sang Won Suh

**Affiliations:** 1 Department of Physiology, College of Medicine, Hallym University, Chuncheon 24252, Republic of Korea; dae.ki.hong@emory.edu (D.K.H.); sshlee@hallym.ac.kr (S.H.L.); ttiger1993@gmail.com (B.S.K.); bagmingyu50@gmail.com (M.K.P.); 2 Department of Pathology and Laboratory Medicine, Emory University School of Medicine, Atlanta, GA 30322, USA; 3 Neuroregeneration and Stem Cell Programs, Institute for Cell Engineering, College of Medicine, Johns Hopkins University School of Medicine, Baltimore, MD 21205, USA; arakho136@naver.com; 4 Department of Neurology, Johns Hopkins University School of Medicine, Baltimore, MD 21205, USA; 5 Department of Physical Education, Hallym University, Chuncheon 24252, Republic of Korea; bychoi@hallym.ac.kr; 6 Institute of Sport Science, Hallym University, Chuncheon 24252, Republic of Korea

**Keywords:** transient receptor potential (TRP) channels, zinc, ischemic stroke, epilepsy, traumatic brain injury

## Abstract

Maintaining the correct ionic gradient from extracellular to intracellular space via several membrane-bound transporters is critical for maintaining overall cellular homeostasis. One of these transporters is the transient receptor potential (TRP) channel family that consists of six putative transmembrane segments systemically expressed in mammalian tissues. Upon the activation of TRP channels by brain disease, several cations are translocated through TRP channels. Brain disease, especially ischemic stroke, epilepsy, and traumatic brain injury, triggers the dysregulation of ionic gradients and promotes the excessive release of neuro-transmitters and zinc. The divalent metal cation zinc is highly distributed in the brain and is specifically located in the pre-synaptic vesicles as free ions, usually existing in cytoplasm bound with metallothionein. Although adequate zinc is essential for regulating diverse physiological functions, the brain-disease-induced excessive release and translocation of zinc causes cell damage, including oxidative stress, apoptotic cascades, and disturbances in energy metabolism. Therefore, the regulation of zinc homeostasis following brain disease is critical for the prevention of brain damage. In this review, we summarize recent experimental research findings regarding how TRP channels (mainly TRPC and TRPM) and zinc are regulated in animal brain-disease models of global cerebral ischemia, epilepsy, and traumatic brain injury. The blockade of zinc translocation via the inhibition of TRPC and TRPM channels using known channel antagonists, was shown to be neuroprotective in brain disease. The regulation of both zinc and TRP channels may serve as targets for treating and preventing neuronal death.

## 1. Introduction

### 1.1. Overview: Transient Receptor Potential Channels in the Brain

The transient receptor potential (TRP) channels consist of six subfamilies with a total of twenty-eight members. Subfamily includes the canonical (TRPC), melastatin (TRPM), vanilloid (TRPV), ankyrin (TRPA), mucolipin (TRPML), polycystic (TRPP) and NOMPC-like (TRPN, possess only invertebrate and fish, not in mammals) categories [1,2,3]. Activation of these TRP channels through external or internal stimuli depolarizes membrane action potential. Altered action potential induces an influx of calcium or zinc ions through voltage-gated ion channels [4,5]. These channels are all highly expressed in the brain and regulate post-synaptically bound receptors and channels such as the N-methyl-D-aspartate receptor (NMDAR), voltage-gated calcium channel, and acid-sensing ion channel (ASIC) [5,6,7]. All TRP channels consist of six putative transmembrane spanning domains (S1–S6) that form a loop shape between the S5 and S6 domains [8,9]. Under physiological conditions, these channels regulate neuronal and glial functions, including signal transduction, action potential, development, and homeostasis [10].

The activation of TRP channels by various internal stimuli includes signal transductions and the chemical, biological, and (especially) intracellular storage of calcium concentrations [11]. The alteration of cytosolic calcium concentrations through TRP channels plays especially diverse fundamental roles in the synaptic-vesicle exocytosis-induced release of neurotransmitters, cell proliferation, and cell death [12,13]. Some TRP channels regulate the concentration of diverse cations such as sodium, calcium, magnesium, and zinc [4,14,15,16,17,18]. In this review, we provide current insights and experimental results related to the physiological and pathophysiological roles of zinc entry with other cations through the TRP channels, based on previous reports [19,20,21].

### 1.2. Transient Receptor Potential Canonical Channels (TRPC)

*TRPCs*. TRPCs, which consist of seven subunits (TRPC1–TRPC7), are expressed in most brain regions and calcium-permeable cation channels. Notably, TRPC2 is a pseudogene in humans and will not be discussed in this review [7]. The general roles of TRPCs include regulating progenitor cell proliferation and enhancing neural survival affected by neurotrophin-like brain-derived neurotrophic factor (BDNF) and neuronal growth by guiding neurite outgrowth [22,23].

1. *TRPC1*. The activation of TRPC1 is initiated by metabotropic glutamate receptors (mGluR1) in neurons, and the formation of excitatory post-synaptic potential is due to TRPC1 functions [11,24,25]. TRPC1 also impacts the developing brain by directing axonal growth [22,26]. In a previous study, short hairpin RNA (shRNA)-induced knockdown of the TRPC1 gene revealed that the adult neural progenitor cell cycle in the G1 phase was significantly decreased [27].
2. *TRPC3*. TRPC3 is also abundantly expressed in the brain, especially in the hippocampus. The main function of TRPC3 is slow excitatory post-synaptic potential (EPSP) and the progression of long-term depression (LTD) [28,29]. The function of TRPC3 is similar to that of TRPC1 which has different expression patterns (approximately ten times that of TRPC3). In addition, TRPC3 gene deletion was related to greater motor deficits compared with the wild type, but there were no differences in brain development [28]. TRPC3 is activated by BDNF, an essential neurotrophic factor for neuronal differentiation, maturation, and survival that possibly interacts with tropomyosin receptor kinase B [30,31,32].
3. *TRPC4* and *TRPC5*. The similarity between TRPC4 and TRPC5 is due to these genes being homologs. Indeed, the TRPC4 and TRPC5 proteins share amino acid sequences with an approximately 65 percent overlap [33]. The structural N-terminal (S1 domain of the TRP channel) coil interacts with regulating microtubule dynamics and stathmin [26]. TRPC4 and TRPC5 are activated by Gαi, a G-protein-coupled receptor, and implicated in neuronal neurite growth and branching problems [34]. These findings suggest that TRPC4 and TRPC5 are closely associated with the neuronal developmental pathways. Although the structures and functions of TRPC4 and TRPC5 are similar, TRPC5 is activated by oxidative damage, and the early induction of a calcium influx especially increases zinc [35].
4. *TRPC6*. TRPC6 is distributed in the molecular layer of the dentate gyrus and tyrosine–hydroxylase-positive neurons in the substantia nigra [36,37]. The molecular structure of TRPC6 shares approximately 75% of amino acids with TRPC3 and TRPC7 (homomultimers or heteromultimers) [6,38]. A previous study demonstrated that the overexpression of TRPC6 increases the numbers of branches and spines in hippocampal neurons, while TRPC6 knockdown using RNA interference decreases these counts [39]. These results suggested that TRPC6 is related to functions of learning, memory, and brain plasticity and confirmed that TRPC6 promotes dendritic growth via the calcium/calmodulin-dependent kinase 4-cAMP response element-binding protein (CREB) signaling pathway [40].

### 1.3. Transient Receptor Potential Melastatin (TRPM)

*TRPMs*. TRPMs consist of eight subunits (TRPM1–TRPM8) and are ubiquitously expressed in tissues, including several brain regions. TRPMs are highly permeable to cations including calcium, magnesium, and zinc, which easily pass and are implicated in neuronal plasticity [4,16,41]. The N-terminal regions of TRPM (S1 domain) consist of four melastatin homology regions that have roles in sensing external stimuli and channel assembly [42,43]. The C-terminal regions of TRPM (S6 domain) are composed of the coiled-coil domain, which regulates the pore gating process [44].

5. *TRPM1* and *TRPM3*. The molecular structures of TRPM1 and TRPM3 are similar in that these channels share about 75 percent of their amino acid sequences [45]. TRPM1 is mainly expressed in the retinal region, and the corresponding knockout mouse presented impaired visual function [46]. TRPM3 is expressed in brain regions involving the hippocampus, cortex, cerebellum, and choroid plexus, especially the somatosensory neurons that regulate inflammation [47,48].
6. *TRPM2*. TRPM2 is highly expressed in the brain, especially in the hippocampus, stratum, and cortex, where TRPM2 is preferentially distributed in the microglia and macrophages [49,50,51]. As a non-selective cation channel, TRPM2 is permeable to diverse cations involving calcium and magnesium through the S5 to S6 domains [52,53]. The activation of TRPM2 is triggered by inflammatory cytokines, tumor necrotic factor alpha (TNF-α)-mediated calcium overload, and cell damage cascades [54]. This channel can also be activated by the intense stimulation of NMDAR and external stimuli that depend on reactive oxygen species (ROS) and hydrogen peroxide via intracellular poly ADP-ribose (ADPR) [55,56,57,58]. One pivotal role of TRPM2 is as an oxidative stress-induced redox signaling sensitive cation-permeable channel [59]. The antioxidant glutathione has an inhibitory effect on the current of TRPM2 in primary cultured neurons through a thiol-independent mechanism [60]. LTD impairment was shown in TRPM2 knockout mice, which significantly decreased expression of the major regulator protein of excitatory synapses, post-synaptic density protein and the α-amino-3-hydroxy-5-methyl-4-isoxazolepropionic acid (AMPA) receptor [41].
7. *TRPM4* and *TRPM5*. TRPM4 is ubiquitously expressed in the heart, prostate, and other tissues, while TRPM5 is primarily found in sensory receptor cells [61,62]. Both have molecular similarities in their sequence homology (about 50 percent) and share properties related to voltage-dependence, single-channel conductance, and channel regulation [62,63].
8. *TRPM7*. The N-terminal of TRPM7 contains a serine–threonine alpha-kinase domain [64,65]. The alpha-kinase domain on the S6 region of TRPM7 regulates cell growth and proliferation via eEF2-kinase/eEF2 or Akt/mTOR signaling [66,67]. TRPM7 is also a non-selective diverse cation channel that is permeable to zinc, magnesium, and calcium (order of permeability: zinc > magnesium > calcium) [68]. TRPM7 characteristics make it a target for brain injuries which activate oxidative stress [69] and also make it highly permeable to calcium and zinc. The physiological functions of TRPM7 are closely related in neurotransmitter release [70], proliferation, and cell survival [67,71]. TRPM knockdown using shRNA in animals showed significantly decreased synaptic density, memory function, and long-term potentiation under physiological conditions [72].

### 1.4. Transient Receptor Potential Vanilloid (TRPV)

*TRPV1.* The TRPV subfamily consists of six subunits (TRPV1–TRPV6) and these channels have high permeability to calcium ion. In the brain, especially, TRPV1 is activated by diverse exo- and endogenous stimuli involving biotoxins and capsaicin [73,74]. Physiological roles of TRPV1 are related to neural development and neurogenesis in the hippocampal subgranular and subventricular zones which are active regions of neurogenesis [75]. Previous studies have reported that the TRPV1 agonist dihydrocapsaicin (DHC) attenuates ischemic stroke-induced cortical injury through hypothermic effect [76,77,78]. TRPV1 expression is also increased in the hippocampal dentate gyrus after temporal lobe epilepsy [79]. TRPV1 antagonists, capsazepine or 5′-Iodoresiniferatoxin, have potential therapeutic use in preventing excessive calcium entry and decreasing pain [80,81,82].

### 1.5. Physiological and Pathological Properties of Zinc in the Brain

Zinc is an essential cation abundantly distributed in all animal tissues, especially in the central nervous system (CNS) and hippocampus. Most zinc is tightly bound to metalloproteins and present in the metallothionein form or free ionic form (Zn^2+^) within the synaptic vesicles along with several neurotransmitters such as glutamate [83,84]. Loosely bound zinc or Zn^2+^ is found in the synaptic vesicles and released by external or internal stimuli to the synaptic cleft. Re-uptake occurs in nearby synaptic terminals for signal transduction [85,86]. Synaptically located zinc is highly enriched in hippocampal mossy-fiber terminals. In contrast, other CNS regions including the cerebellum and spinal cord contain low concentrations of zinc [87]. Adequate concentrations of zinc are critical under physiological conditions, as zinc is essential for cell division and DNA synthesis, as well as to establish memory function [88,89]. However, in the case of brain diseases such as hypoglycemia, head trauma, epilepsy, and ischemic stroke, it was found that excessively released zinc from synaptic vesicles is translocated and accumulated in the post-synaptic neurons of the hippocampal pyramidal layer and dentate gyrus, as visualized by N-(6-methyl-8-quinolye)-para-tolurnesulfonamide [90]. Zinc has bilateral roles in both physiological and pathophysiological conditions, acting as a “double-edged sword”.

Zinc transporters (ZnTs), as well as Zrt- and Irt-like proteins (ZIPs), mediate Zn^2+^ transport from the cytosol to cell organelles or extracellular space. Most members of the ZnT subfamily are ubiquitously located and expressed in cellular organelles, including synaptic vesicles and the plasma membrane [91]. ZnT1 is expressed in the dendritic spine and synaptic membrane [92] and was implicated in Zn^2+^ overload-induced neuronal and glial damage, as well as brain disease [93,94]. The primary role of ZnT3 is regulating vesicular zinc. Vesicular zinc was reported to be implicated in hippocampal neurogenesis and cognitive function [95]. In a previous study, ZnT3 knockout-induced vesicular zinc depletion significantly reduced adult hippocampal neurogenesis, and supplementary zinc treatment remarkably enhanced progenitor cell proliferation and cognitive function [85]. An autistic phenotype was also observed in ZnT3 knockout mice and significantly decreased social interaction and novelty preference. The ZnT3 knockout brain was found to be larger than that of the wild type (megalencephaly) due to overexpressed BDNF-induced neurite outgrowth and increased neurogenesis [96].

Zinc plays an essential role in maintaining homeostasis, including the neurotransmitter and neuromodulator regulating cycle and synaptic plasticity [97,98]. Zinc homeostasis is destroyed by overloaded cellular zinc due to diverse brain diseases including ischemia, head trauma, and epilepsy. Brain-injury-induced excessive release of endogenous zinc at the glutamatergic postsynaptic neurons exacerbates pathogenesis. Zinc released by synaptic activity can cross the plasma membrane that is regulated by diverse membrane-bounded channels involving the NMDAR, the glutamate receptor 2 (GluR2)-lacking AMPA/kinase channel, the voltage-gated calcium channel, and the sodium–calcium exchanger [99,100]. The pathological properties of zinc in the brain are complex and complicated in that increased zinc can be deleterious to neurons [101] and induce apoptotic damage [102]. Increased intracellular zinc acts as an ionic regulator of excitotoxic neuronal damage [90,103], and brain-damage-mediated oxidative stress disassembles the loosely bound zinc from metalloproteins (zinc-binding proteins), which are an essential means of maintaining the zinc level in the brain; this leads to an increasing zinc concentration [104,105]. Intracellular organelles involving mitochondria, lysosomes, and the endoplasmic reticulum excessively take up and store the increased intracellular zinc, which induces ROS production; consequently, the ROS-induced oxidative damage triggers cell death [106,107,108]. One of the possible aggravating cascades of zinc is overloaded zinc in the mitochondria. This causes caspase-dependent apoptotic cascades through depolarization, and the cytochrome C release via mitochondrial zinc contributes to the mitochondrial permeability transition pore [109,110]. In addition, the lysosomal zinc concentration rises rapidly after external or internal stimuli such as hydrogen peroxide exposure. Lysosomal zinc constructs a lysosomal membrane; consequently, proteolytic factors are secreted to the cytoplasm and lead to cell death [105].

However, pathological zinc-overloaded brain and spinal cord damage was attenuated by ZnT3 knockout: (1) The intra-hippocampal injection of colchicine induced extensive neuronal death, disturbance of axonal transport, destruction of the cytoskeletal structure, and zinc accumulation. However, in ZnT3 knockout mice, colchicine-injection-induced brain damage was strikingly attenuated [111]. (2) Myelin oligodendrocyte glycoprotein 35–55 peptide, *Mycobacterium tuberculosis*, and pertussis toxin-induced experimental autoimmune encephalomyelitis (EAE) caused neuronal demyelination, monocyte and neutrophil infiltration, immunoglobulin extravasation, and zinc accumulation. Vesicular zinc depletion via ZnT3 knockout reduced EAE-induced spinal cord damage [112]. On the other hand, from the perspective of clinical trials, chemotherapy can induce cognitive impairment. The anti-cancer agent, paclitaxel, decreases vesicular zinc concentration and hippocampal neurogenesis during the acute and chronic treatment period. Supplementing zinc can attenuate chemotherapy (paclitaxel)-induced cognitive dysfunction [113].

Several studies have explored targeting excessively released zinc from synaptic vesicles in neurological diseases to reduce brain damage. The zinc-chelating agent (1) extracellular zinc chelator, clioquinol (5-chloro-7-iodo-8-hydroxy-quinoline), has protective effects against EAE-induced neuronal demyelination and spinal cord damage [114]. (2) Targeting zinc and glutathione using N-acetyl-l-cysteine (NAC) attenuates hypoglycemia-induced neuronal damage cascades, including zinc overload [115]. (3) Targeting zinc and AMPK using the novel developed compounds 1H10 and 2G11s in EAE and ischemic stroke reduced spinal cord [116] and brain damage [117]. 

Following the above zinc-related published studies, this review considered the diverse modulatory roles of zinc and the TRP channels in pathophysiological conditions in the brain. Diverse divalent cations move into the intracellular space through most TRP channels, which are also zinc permeable (Table 1).

Previous studies have shown that brain injuries such as ischemia-, TBI-, epilepsy-, or hypoglycemia-induced vesicular zinc release, or liberation of free zinc from zinc-binding metalloproteins, cause neuronal death cascades such as ROS production, glial activation and apoptosis. These cascades are closely related to additional TRP channel activation and leading to dysfunction of ionic homeostasis and aggravation of zinc accumulation [7,120,124]. Next, we provide that regulation of TRP channels especially TRPM and TRPC can contribute neuroprotection in diverse brain injuries such as cerebral ischemia, epilepsy, and traumatic brain injury through antagonists of TRPM and TRPC based on previous studies.

## 2. TRP Channels and Zinc in Brain Disease

### 2.1. Cerebral Ischemia

**Figure 1:**
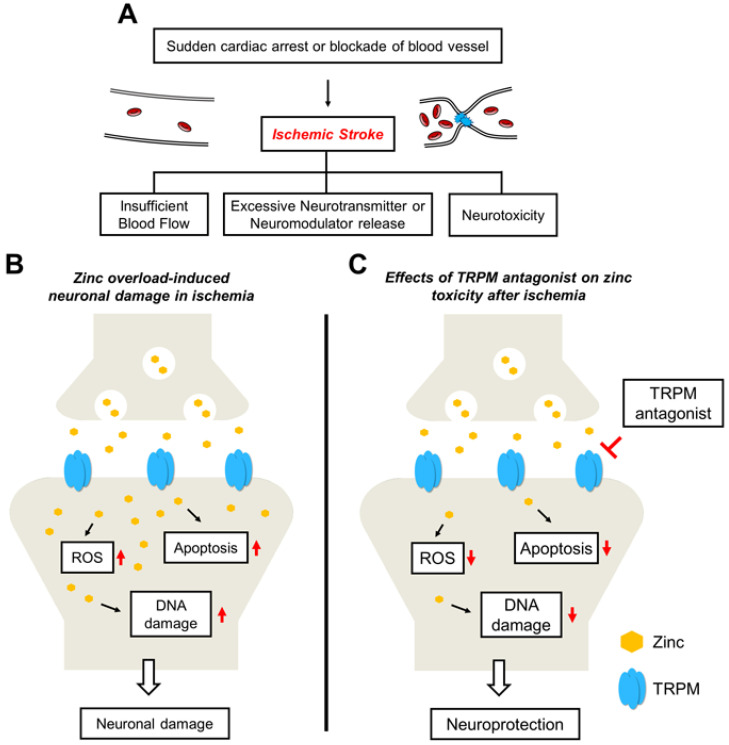
Proposed cascades for the neuroprotective effects of TRPM antagonists on global cerebral-ischemia-induced neuronal death. (**A**) The main cause of brain ischemia is drastically halted blood flow and the blockage of blood vessels. Deleterious mechanisms following brain ischemia are neurotoxicity, glutamate excitotoxicity, and zinc overload. (**B**) A schematic drawing illustrating the possible cascades via ischemia that can induce neuronal damage. Zinc that is excessively released from pre-synaptic vesicles and accumulated through the TRPM channel can trigger ROS production, thereby damaging the apoptotic pathway and DNA. Consequently, this accumulation can contribute to neuronal damage. (**C**) Possible neuroprotective properties of TRPM antagonists. Reduced post-synaptic zinc entering through the TRPM channel via antagonists can reduce neuronal damage.

Ischemic stroke is caused by various injuries in which a sudden cardiac-arrest-induced decrease in the systemic blood flow or the blockage of brain vessels by blood clots causes a cessation of brain perfusion. There is an extreme risk of neurological impairment including cerebral infarction, neuronal loss, and behavioral dysfunction in global cerebral ischemia (GCI) survivors following the return of spontaneous circulation [125,126]. Post-resuscitation or in the thrombolytic phase after a GCI, additional ischemic injuries through blood reperfusion underlying pathophysiological cascades can cause brain damage through, e.g., free radical formation, neuroinflammation, and altered ionic homeostasis [127,128] (Figure 1A). The main ischemic problems are excessive neurotransmitter and neuromodulator release from the synaptic vesicles, including glutamate, calcium, and zinc, causing influxes to nearby postsynaptic neurons through a cation-selective channel. Overloaded calcium via NMDAR was inhibited by increasing the TRPC6 in transgenic mice [129]. Inhibited TRPC6 degradation by hyperforin following ischemia activated the neuroprotective pathway involving the CREB pathway to reduce brain damage [130]. One ischemic category, intracerebral hemorrhage (ICH), is an intracranial bleeding stroke. ICH leads to the formation of a glial scar that causes tissue damage, neuroinflammation, and toxic edema. The provoked neuroinflammatory cytokines, such as neutrophils, macrophages, and chemokines, lead to secondary injury following the primary ICH [131]. Thrombin in the hematoma treatment in cultured astrocytes activated TRPC3 expression [132], and the TRPC3 inhibitor, Pyr3, reduced astrocyte activation and behavior impairments following an ICH [133]. These reports suggest that diverse TRP channels are closely associated with cerebral ischemia, and targeting the TRP channels may be a therapeutic window for ischemic problems. In addition, Hong et al. developed a novel zinc chelator, 2G11, based on an AMPK inhibitor, compound C, through virtual screening [117]. AMPK is a well-known energy metabolism regulatory factor; however, the high phosphorylation of AMPK has deleterious effects on neurons, which activates neuronal nitrous oxide synthase (nNOS), and zinc excitotoxicity induces pre-apoptotic factors such as LKB1 and Bim [134,135].

Previous studies have confirmed high accumulations of extracellular zinc in the intraneuronal space in cerebral ischemic animal models [136]. There are possible deleterious cascades of overloaded zinc affecting the surrounding neurons or cellular organelles in an ischemic condition. Extra- and intraneuronal zinc chelation by N,N,N′,N′-tetrakis(2-pyridylmethyl)ethylenediamine) OG significantly reduced brain infarction, behavior impairments, apoptotic cell damage including poly(ADP-ribose)polymerase-1 (PARP-1) cleavage, and PAR accumulation [137]. Overloaded zinc causes PARP activation, which is an essential modulator for cell damage cascades from diverse stimuli. In addition, zinc chelation in the ischemic condition contributed to decreasing ROS production, and the inhibition of NADPH oxidase-derived ROS production decreased zinc translocation [138]. These previous studies suggest that zinc is closely associated with neuronal-death-inducing cascades, and zinc translocation might be accelerated via zinc-related membrane-bounded channels from the initiated cell-death process (Figure 1B).

TRPM7 has been implicated in the transport of cations such as calcium, magnesium, and zinc. Aarts et al. demonstrated that oxygen–glucose deprivation-induced calcium overload and neuronal death were attenuated by using TRPM7 siRNA [69]. Sun et al. found that the recombinant adeno-associated virus-mediated suppression of hippocampal TRPM7 offered neuroprotection against global ischemia [139]. These results suggest that TRPM7 is closely related to brain injury, especially ischemic stroke. Several studies have investigated the relationship between TRPM7 and zinc. Inoue et al. showed that an overdose of extracellular zinc contributed to the activation of TRPM7 and increased intracellular zinc accumulation in cultured cells. Moreover, knockdown of TRPM7 using TRPM7 shRNA decreased zinc neurotoxicity [120]. Hong et al. applied known inhibitor of the TRPM7 channel, carvacrol (extracted from *Origanum vulgare*, a monoterpenoid phenol) [140,141], to a GCI animal disease model. It is known that carvacrol also activates TRPA1 and TRPV3 [142,143]. GCI was found to induce extensive neuronal death, zinc accumulation from excessively released pre-synaptic vesicles, oxidative-damage-mediated lipid peroxidation, and microglial and astroglial over-activation. Specifically, the authors observed the results after increasing TRPM7 activation three days after GCI. Carvacrol was found to offer neuroprotective effects against GCI by decreasing the expression of TRPM7 and zinc accumulation [144] (Figure 1C).

### 2.2. Epilepsy

**Figure 2:**
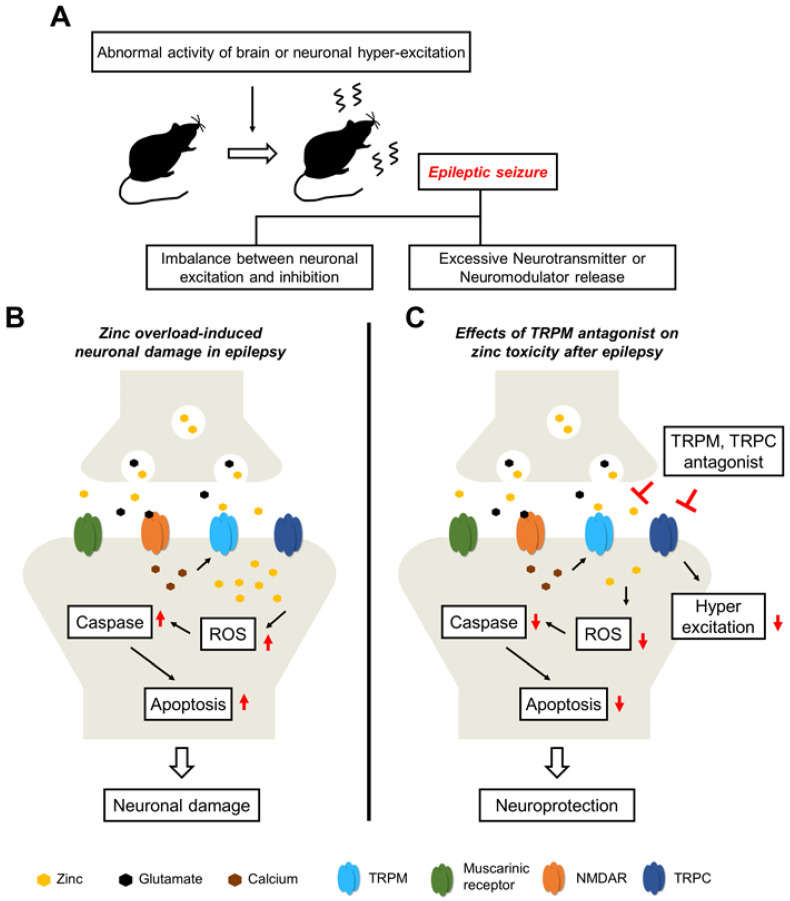
Proposed cascades for the neuroprotective effects of TRPM antagonists on epileptic seizure-induced neuronal death. (**A**) The main cause of epileptic seizures is excessively released excitatory-neurotransmitter-induced hyper-excitation and the dysfunction of neuronal action potential. (**B**) Schematic drawing showing how the possible cascades via epileptic seizures induce neuronal damage. Zinc that is excessively released from pre-synaptic vesicles and accumulated through the TRPM channel can trigger ROS production and lead to the apoptotic pathway, thereby contributing to neuronal damage. (**C**) Possible neuroprotective properties of TRPM antagonists. Reduced post-synaptic zinc entering through the TRPM channel via antagonists can reduce neuronal damage.

Epilepsy is characterized by a lasting predisposition to experiencing spontaneous epileptic seizures and suffering from unpredictable seizure behaviors [145,146]. The primary mechanism that initiates epileptic seizures is not understood, but diverse types of brain damage such as hypoxia, head trauma, and stroke contribute to promoting epileptic problems [147,148]. The signaling cascades in epilepsy are primarily caused by the disturbance of the ionic homeostasis, and the neural circuits show highly increased excitation [149] (Figure 2A). Although several risk factors can exacerbate epileptic brain damage, previous studies have focused on highly accumulated, zinc-mediated neurotoxicity [150,151,152] (Figure 2B). Epileptogenesis is closely related to greater depolarization in neurons and a high susceptibility to epileptic activity; the blockade of TRPC3 contributes to diminishing these hyper-excitable effects [153]. Epilepsy induces the downregulation of TRPC6, which increases seizure susceptibility and neuronal polarization. TRPC6 knockdown inhibited the programed cell death in the hippocampus following status epilepticus (SE) [154].

A cascade of epileptic-seizure-induced hippocampal neuron death is also associated with zinc concentration. ZnT3 null mice with kainate-induced epilepsy displayed decreased neuronal loss in the hippocampal CA1 region in the acute phase [155], and the ZnT3-reflected absence of vesicular zinc had more sensitivity following the epileptic seizure score [156]. However, a chronic zinc supplementation diet in an epileptic seizure animal model restored the cognitive and behavior outcomes, abnormal expression of GPR39, ZnT3, and myelin basic protein (MBP) when compared with a zinc-deficient diet [157]. These results suggest that consistent zinc supplementation induces positive outcomes after a seizure, but in the early-time phase of a seizure, modulating zinc concentration may be a therapeutic window for epileptic neuronal damage.

Khalil et al. demonstrated that carvacrol treatment after electrode-implantation-induced recurrent SE protected against cognitive decline, hippocampal neuronal damage, and early seizure frequency [158]. Jeong et al. applied the TRPM7 antagonist, carvacrol, to a pilocarpine-induced epileptic seizure animal model. Epilepsy causes the overexpression of TRPM7, excessive zinc accumulation, oxidative damage, immunoglobulin extravasation, and neuronal death. Carvacrol has overall neuroprotective effects on all aspects of epilepsy except for seizure-grade epilepsy (Racine stage). They also used 2-aminoethoxydiphenyl borate (2-APB), known nonspecific TRP-channel modulator, 2-APB inhibits TRPM7 [159] and TRPM2 [160] and also activates a number of TRPV channels [161,162]. Our previous study focused on the inhibitory effect of 2-aminoethoxydiphenyl borate (2-APB) on TRPM7 and found that it significantly reduced TRPM7 overexpression, zinc translocation, and neuronal damage [163] (Figure 2C).

### 2.3. Traumatic Brain Injury

**Figure 3:**
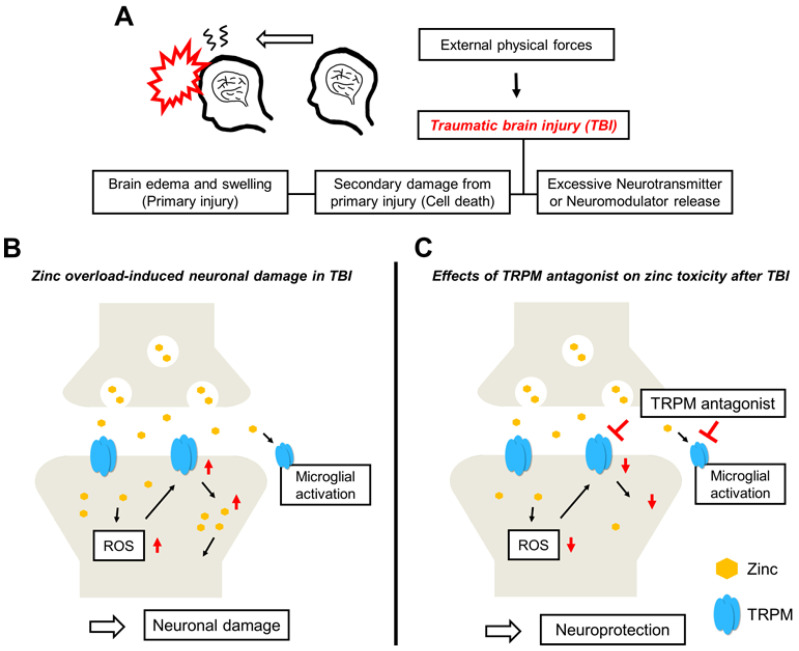
Proposed cascades for the neuroprotective effects of TRPM antagonists on traumatic brain-injury-induced neuronal death: (**A**) The main causes of TBI are external physical forces and stimuli. TBI-induced primary damage produces brain edema, and swelling is caused instantly. Secondary damage from primary insult leads to deleterious cellular cascades. (**B**) Schematic drawing showing how the possible cascades via TBI induce neuronal damage. Excessively released zinc from pre-synaptic vesicles and zinc accumulated through the TRPM channel can trigger ROS production and, thereby, trigger further activation of TRPM, leading to an increase in the intracellular zinc concentration. This concentration of zinc contributes to neuronal damage. (**C**) Possible neuroprotective properties of TRPM antagonists. Reduced post-synaptic zinc entering through the TRPM channel via antagonists can reduce neuronal damage.

Traumatic brain injury (TBI) is caused by external forces, such as accidents and violence, and causes disabilities in millions of people. TBI is a devastating brain injury that contributes to neuronal loss and the subsequent dysfunction of memory and cognition. When head-impact-induced primary injury occurs, the resulting immediate TBI can include brain edema and swelling that does not immediately cause neuronal damage. However, TBI evolves afterwards [164]. Secondary injury almost always follows the primary injury and can lead to severe brain damage, especially zinc translocation and microglial activation [165,166] (Figure 3A,B). There are several lines of evidence for suggesting a therapeutic approach following TBI-induced neuronal death using hypothermia, applying antioxidative compounds, and reducing the vesicular zinc [167,168,169]. Following TBI, there are increases in the TRPM2 mRNA levels in the hippocampal and cortical regions [170]. Vehbi et al. reported that TRPM2 reduction using melatonin decreased oxidative stress, apoptotic damage, and calcium influx after TBI [171].

Zinc is highly accumulated in postsynaptic neurons and causes neurotoxicity in the brain. Pretreatment of the extracellular zinc chelator, calcium ethylenediaminetetraacetic acid, increased the neuroprotection-related gene expression, including heme oxygenase-1 (HO-1), glutathione peroxidase 1 (GPX-1), and heat-shock protein 70 (HSP-70), and decreased apoptotic cell death following TBI [172]. However, a zinc-deficient diet contributes to the disturbance of hippocampal neurogenesis following TBI, where progenitor cell proliferation is immediately increased after a brain injury because of the repair process during the compensative mechanisms [173]. Choi et al. reported that the metal chelator, 5-chloro-7-iodo-8-hydroxy-quinoline (Clioquinol, CQ), reduced progenitor cell proliferation, neuroblast production, and vesicular zinc distribution, reducing TBI-induced neuronal damage [174].

Li et al. demonstrated the neuroprotective effects of carvacrol on cortical neurons with TBI. Increased intracellular calcium concentrations through TRPM7 were attenuated by carvacrol treatment. The results found that carvacrol treatment decreased calcium-overload-induced cellular damage, including neuronal nitric oxide synthase (nNOS) expression [175]. Xu et al. used a controlled cortical impact (CCI)-induced TBI animal disease model. TRPM suppression by shTRPM7 following TBI decreased TBI-induced neurological deficits, apoptotic cell death cascades including terminal deoxynucleotidyl transferase dUTP nick-end labeling (TUNEL) staining, and cleaved caspase-3 expression [176]. Park et al. demonstrated that the TRPC5 antagonist NU6027 has neuroprotective effects on a CCI-induced severe TBI animal model. The authors found that NU6027 treatment after TBI attenuated TBI-induced neuronal damage, oxidative stress, glial activation, and zinc translocation in the hippocampal CA3 region. TRPC5 was known and defined as a calcium channel, but these results suggest that zinc may also translocate through TRPC5 [177]. Lee et al. found that carvacrol decreases TRPM7 expression after TBI and that TBI-induced neuronal damage cascades including zinc accumulation were attenuated by carvacrol with the inhibition of TRPM7. Carvacrol recovered neurological deficits and delayed neuronal loss 7 days after TBI. The authors also demonstrated that the other TRPM7 antagonists 2-APB and NS8593 provide neuroprotective effects after TBI [166] (Figure 3C).

## 3. Conclusions

The process of neurons being damaged after several brain injury types involves diverse extra- and intracellular detrimental cascades, mainly the homeostasis disturbance-induced dysregulation of cellular functions. Recent preclinical studies have found that the physiological and pathological properties of TRP channels and zinc have deleterious effects in brain diseases. This review focused on both alterations in the ionic gradient between extra and intracellular spaces through TRP channels and the translocation of zinc with ionic influxes. Blocking these channels was found to have neuroprotective properties in multiple brain disease conditions and represents a promising therapeutic avenue. Additionally, the regulation of zinc concentration is essential for the prevention of brain injury because zinc overload can contribute to, or exacerbate, brain damage following different types of such injuries. Several reports explain that using zinc as a neurotoxic component in brain disease and regulating zinc concentration levels unveils a therapeutic approach. On the other hand, consistent zinc supplementation recovers behavior outcomes in animal disease models and clinical trials. Therefore, in this review we suggest that modulation of the zinc concentration in each brain disease or time-dependent phase is an effective approach to solving brain injury-induced neuronal damage; moreover, targeting risk factors for these devastating brain disorders could represent an effective option for treating or preventing ischemic, epileptic, and traumatic brain-injury-induced neuronal death.

## Figures and Tables

**Table 1:** Zinc-permeable and sensitivity of TRP channels.

Channels	Experimental Results	References
TRPM2	TRPM2-knockout microglial cells prevented high zinc exposure-induced zinc neurotoxicity. Hydrogen peroxide-induced intracellular zinc accumulation and ROS production were prevented in TRPM2-knockout hippocampal neurons.	[118,119]
TRPM7	TRPM7 is highly permeable to zinc and overexpression of TRPM7 exacerbates zinc toxicity.	[68,120]
TRPA1	Zinc entering through TRPA1 activates TRPA1 and causes pain and irritation via zinc toxicity.	[121]
TRPV6	TRPV6 transports zinc and overexpressed TRPV6 contributes to increasing zinc intoxication.	[122,123]
TRPC5	High zinc exposure-induced cellular toxicity through delayed calcium entry was prevented by TRPC5 blocker.	[35]

## Data Availability

All experimental datasets in the current study are available on reasonable request to the corresponding author.
